# Copper nickel co-impregnation of Moroccan yellow clay as promising catalysts for the catalytic wet peroxide oxidation of caffeine

**DOI:** 10.1016/j.heliyon.2021.e06069

**Published:** 2021-01-31

**Authors:** Ouissal Assila, Morad Zouheir, Karim Tanji, Redouane Haounati, Farid Zerrouq, Abdelhak Kherbeche

**Affiliations:** a Laboratory of Catalysis, Materials and Environment, Higher School of Technology, Sidi Mohamed Ben Abdellah University of Fez, 30000 Fez, Morocco; b Laboratory of Physical Chemistry and Environment (LPCE), Faculty of Sciences, Ibn Zohr University, City Dakhla, Agadir, Morocco

**Keywords:** Heterogeneous catalyst (CuNi-YC), Catalytic wet peroxide oxidation, Impregnation, Caffeine, Response surface methodology

## Abstract

Copper and nickel were incorporated into the prepared yellow clay (YC) using one of the most widely used methods, for the preparation of heterogeneous catalysts, which is the wet impregnation method (IPM) and its application as a heterogeneous catalyst for Caffeine (CAF). Several catalysts Cooper Nickel's Catalysts (Cu–Ni) were applied to the yellow clay with different weight ratio of Cu and Ni, in order to explore the role of both metals during the catalytic oxidation process CWPO. Furthermore, the CuNi-YC catalysts, were characterized by X-ray diffraction (XRD), X-ray fluorescence (XRF), Langmuir's surface area, Brunauer Emmett Teller (BET), scanning electron microscope (SEM) and inductively Coupled Plasma-Atomic Emission Spectrometry (ICP-AES), so as to get a better understanding concerning the catalytic activity's behavior of CuNi-YC catalysts. The optimization of the catalytic activity's effects on the different weight ratios of Cu and Ni, temperature and H_2_O_2_ were also examined, using Box-Behnken Response Surface Methodology RSM to enhance the CAF conversion. The analysis of variances (ANOVA) demonstrates that Box-Behnken model was valid and the CAF conversion reached 86.16%, when H_2_O_2_ dosage was equal to 0.15 mol.L^−1^, copper impregnated (10%) and temperature value attained 60 °C. In addition, the regeneration of catalyst's cycles under the optimum conditions, indicated the higher stability up to four cycles without a considerable reduction in its conversion performance.

## 1 Introduction

In many cases, the aqueous streams caused by pharmaceutically contain organic pollutants such as caffeine (CAF), which is the most commonly used legal drug throughout the world in the form (beverages or combined) [1, 2], and it is toxic and poorly biodegradable. These polluting agents are also in very high concentrations, so that releasing these molecules into the water resources, affects aquatic life and ecosystems beings, adversely [3]. In these cases, it is necessary to use less conventional techniques to remove the pollutants and convert persistent chemicals into environmentally benign compounds, such as advanced oxidation processes (AOPs) like Fenton process, electro catalytic oxidation, photocatalytic oxidation and catalytic wet peroxide oxidation [4, 5, 6, 7, 8]. The oxidation process with H_2_O_2_ using a heterogeneous catalyst is commonly known as catalytic wet peroxide oxidation (CWPO). CWPO is one of the promising methods for the rate of pollutant degradation at mild temperature and pressure conditions [9, 10, 11, 12], providing that, a suitable catalytic system is used, such as catalysts based on natural and pillared clays (PILCs). These materials are porous, developed by molecular design methods, prepared by exchanging the cations located in the interlayer space of clays with large inorganic polyoxo/hydroxo cations [13, 14, 15].

Clay minerals, a large family of aluminosilicates (Si^4+^ and Al^3+^) structures with a variety of chemical composition, structure and surface properties, very reactive materials due to their small particle size, high surface area and adsorption properties [16, 17]. Bentonite, with a layer structure containing a larger amount of mesoporous, has been widely used in the catalysis field. It constitutes an abundant mineral resource and an effective catalyst support because its strong metal support interaction [18, 19, 20]. Their quality depends on several parameters such as color and swelling behavior which are influenced by the crystal chemistry and mineralogical composition [21]. Bentonites consist mainly of montmorillonite, which is a dioctahedral clay of the smectite group with the 2:1 layer linkage [22, 23]. As the previous research demonstrated, most studies focus on using cheaper transition active metals, such as Ni or Cu. Both metals are widely used as catalysts for a variety of processes, their wide usage can be attributed to their several characteristics such as being very strong catalytic, optical, electrical, mechanical and antifungal/antibacterial. Moreover, Copper (Cu) has been used in catalyst based on the activated carbon (AC), nano-zerovalent copper (nZVCu) functionalized hydroxyapatite (HA), alginate [24, 25, 26] and on the perovskite (LaNiO_3_, NaNi_0.9_Cu_0.1_O_3_ and LaNi_0.5_Cu_0_._5_O_3_) [27,28]. Thus, the objective of the present study is to evaluate the potential use of Moroccan yellow clay as a superb natural support of Copper/Nickel catalysts, in order to enhance its catalytic activity using simple impregnation method for degradation of organic pollutants in aqueous solution [29, 30, 31].

The Response Surface Methodology (RSM) was widely used in many researches for the optimization of different liquid effluent treatment processes. In fact, RSM is a statistical technique applied to reduce the number of experiments, to optimize and analyze the experimental independent parameters that affect the process' efficiency, and to generate a mathematical model, which describes the processes' behavior. Central composite, Doehlert, and Box–Behnken are three classes of response surface designs. Yet, Box–Behnken design is more advantageous because it creates an experimental design with a few test runs, which makes the experiments economically feasible and beneficial [32, 33]. As far as we know, and according to the extensive literature review, CWPO of CAF onto CuNi-YC was not fully investigated. Therefore, the objective of this work is to evaluate the yellow clay extracted from the North of Morocco, exhibit its characterization as a natural eco-friendly and low-cost material and highlight its availability and usefulness when it will be modified using Nickel and Copper by impregnation method. Thus, work will allow the determination of its physicochemical properties and then the identification of its field of use as a catalyst for CWPO, which has never been studied before. In order to examine its effectiveness in oxidation after its modification, the caffeine (CAF) molecule was used in this study as a persistent and hardly removable pollutant to be removed from aqueous solution in a batch reactor. Furthermore, the optimization of degradation's efficiency is an interesting study; hence, a response surface methodology based on Box–Behnken Design (BBD) was used with a three-level factorial design, to optimize the effects of three significant factors: impregnated copper (%), temperature (20–60 °C) and H_2_O_2_ dosage (8.2∗10^−2^ – 24.6∗10^−2^ mol.L^−1^) which influence the CWPO process.

## 2 Materials and methods

### 2.1 Catalysts preparation

The following chemicals were used in the catalysts’ preparation (CuNi-YC samples): Copper (II) Nitrate Hexahydrate (Cu (NO_3_)_2_.6H_2_O, Sigma-Aldrich, 99.99% purity), Nickel (II) Nitrate Hexahydrate (Ni (NO_3_)_2_.6H_2_O, Sigma-Aldrich, 99.99% purity), Hydrochloric Acid (37%, w/w), Sodium Hydroxide 97% (NaOH) and Hydrogen Peroxide (30%, w/w) Sigma-Aldrich. The Caffeine (C_8_H_10_N_4_O_2,_ Sigma-Aldrich) were used as molecule models for the degradation by catalytic wet peroxide oxidation (CWPO) tests. All chemical materials were used without further purification. The deionized water has been used throughout the experiments.

The catalysts support is referred to as yellow Clay (YC). The one used in this work has been taken from a natural basin of the Tidiennit massif in the North of Morocco. Fraction up to 63 μm. Cu–Ni samples were synthesized by the wet impregnation method where Cu(NO_3_)_2_•6H_2_O, and Ni(NO_3_)_2_•6H_2_O were mixed to obtain the following Cu:Ni weight ratios: 1:0, 1:1 and 0:1 and the obtained catalysts were denoted as CuNi10-YC, CuNi11-YC and CuNi01-YC, respectively. Every solution should contain as much as metal nitrate to get 10 wt% metal in the final catalyst powder. In this process, 10 wt% metal was dissolved in 50 mL of deionized water. Then, the YC was dropped into this aqueous solution with stirring speed of 200 rpm at 75 °C for 4 h, to obtain a slurry. After impregnation, the slurry was dried at 100 °C overnight, and then calcined at 500 °C for 4 h.

### 2.2 Experimental conditions for catalytic runs

In order to evaluate CAF mineralization using the catalysts (CuNi10-YC, CuNi11-YC and CuNi01-YC), an amount of 1 g.L^−1^ of each catalyst was added to 100 mL CAF solutions with a concentration of 40 mg.L^−1^; then, stirred to maintain a uniform suspension. Before starting the CWPO reaction, the adsorption of CAF by the catalysts was performed until reaching the equilibrium, which happen to be at 15 min. This step is a controlling experiment to compare between adsorption of CAF and the CWPO conversion; on the other hand, to insure that the decrease of CAF concentration is attributed to CWPO conversion; then, CWPO reactions were started for each catalysts by adding H_2_O_2_ to the solutions respecting Table 1. After 120 min of reaction, the solution was centrifuged to remove particles; then, analyzed using UV–vis spectrophotometer VWR UV-6300PC at λ_max_ of 272 nm. The degradation efficiency of CAF was evaluated in Eq. (1); Where C_0_ and C_t_ are CAF concentrations (mg.L^−1^) at the time of withdrawal [34, 35, 36, 37, 38].

(1) $$\text{CAF}\;\text{Conversion }\left(\text{\%}\right)=\left[\frac{{\text{C}}_{0}-{\text{C}}_{\text{t}}}{{\text{C}}_{0}}\right]\times 100$$

**Table 1:** Factors and levels used for Box-Behnken Design for the degradation of CAF.

Factors	Symbol	Levels		
		-1	0	+1
**[**H_2_O_2_**]** (mol.L^−1^)	X_1_	0.082	0.164	0.246
Impregnated copper (%)	X_2_	0	5	10
Temperature (°C)	X_3_	20	40	60

The reaction of H_2_O_2_ with the CAF was also carried out for comparison. The total organic carbon (TOC) analyses were determined using an analyzer (TOC-VCSN, Shimadzu) at the end of each reaction to investigate the total mineralization of the CAF in the solutions. The TOC values after 2 h of CWPO reaction were calculated using Eq. (2) [4]. Both, TOC measurements and analytical determinations of CAF concentrations were performed at least twice in order to ensure reproducibility of the measurements.

(2) $$\text{TOC}\left(\text{\%}\right)=\left[\frac{{\text{TOC}}_{\text{i}}-{\text{TOC}}_{\text{f}}}{{\text{TOC}}_{\text{i}}}\right]\times 100$$

The adsorption of Methylene blue dye on raw clay, CuNi10-YC, CuNi11-YC and CuNi01-YC was performed and found to be following Langmuir adsorption isotherm with a monolayers capacity of 30.40 mg.g^−1^, 69.12 mg.g^−1^, 63.54 mg.g^−1^, 58.06 mg.g^−1^ respectively. As methylene blue was reported to have flatwise adsorption from water with effective area per molecule on the surface of 130 Å^2^ [39,40]. Therefore, Langmuir specific surface area has been calculated instead of BET using N_2_, because it reflects a better interpretation of the effective surface area when the adsorption of CAF is carried out from water solution. The following equation was used (3), Where X is the monolayers capacity mentioned above for each catalyst in moles per gram; N is Avagadro number (6.019∗10^23^ mol^−1^) and A is the area of methylene blue molecule.

(3) $$\text{Specific surface area }\left({\text{SSA m}}^{2}.{\text{g}}^{-1}\right)={\text{X}}_{\text{m}}.\text{N}.\text{A}$$

### 2.3 Experimental design

Box-Behnken Design (BBD) was used for the experimental design of the CAF degradation using CuNi-YC, in order to investigate the effect of the main parameters: [H_2_O_2_], catalyst, and temperature with X_1_, X_2_ and X_3_ are the studied coded variables which are calculated by Eq. (4). The independent factors were studied at three different levels, low (−1), medium (0), and high (+1). The predicted response (Y) fitted by second-order polynomial equation is the most commonly used (5), where Y is the measured response, β_0_ is the intercept parameter; β_i_, β_ii_, and β_ij_ represent the linear effects, the quadratic effects, and the interaction effects, respectively. X_i_ and X_j_ are the studied factors. K is the number of the optimized factors and ε is the random error. Hence, NemrodW software was used to process and design the experiments data Table 1 [32, 33].

(4) $$x_{i}=\frac{X_{i}-X_{i,0}}{\text{Δ}X_{i}}\;\left(i=1,2,3\right)$$

(5) $$Y=\beta_{0}+\sum_{i=1}^{k}\beta_{i}X_{i}+\sum_{i=1}^{k-1}\sum_{j=2}^{k}\beta_{ij}X_{i}X_{j}+\sum_{i=1}^{k}\beta_{ii}X_{i}^{2}+\varepsilon$$

The analysis test of variance (ANOVA) was applied to evaluate the results, where the determination coefficient (R^2^ and adjusted R^2^) and p-value (probability) (p < 0.05), are the main parameters used to evaluate the effectiveness, the statistical significance and the prediction capability of the model [41, 42].

### 2.4 Characterization techniques

Plasma-Atomic Emission Spectrometry (ICP-AES) was inductively used to test the Cu and Ni contents in the prepared catalysts using a FR-T-RR-01, CURI. The phase and crystallinity of the catalysts and YC were identified by X-ray diffraction (XRD) using X'Pert Pro PANalytical diffractometer equipped with a detector operating Cu Kα radiation (λ = 1.540598 Å; 40 kV and 30 mA). The X-ray fluorescence (XRF) was used to explore the chemical composition of raw YC. The catalysts' morphology was illustrated by a scanning electron microscopy (SEM) using QUANTA 200 FEI instrument at 30 kV. BET surface area was carried out by N2-adsorption at 77 K using a Micromeritics ASAP 2010 instrument.

## 3 Results and discussion

### 3.1 Characterization results

**Figure 1:**
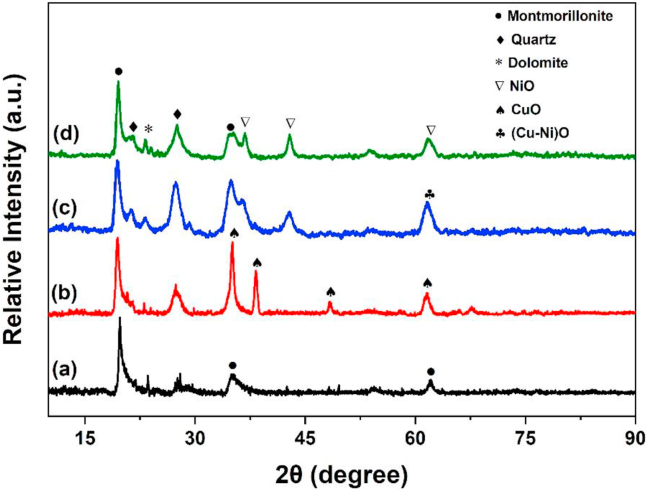
XRPD patterns of the support and CuNi-YC catalysts: (a) support and (b) CuNi 1:0 catalyst; (c) CuNi 1:1 catalyst; (d) CuNi 0:1 catalyst.

The powder X-ray diffraction patterns of the support and synthetized catalysts are shown in Figure 1. In the diffractogram of raw YC Figure 1a, the peaks at 2θ = 19.9°, 35.0°, 61.84° which corresponded to the d_101_, d_107,_ d_060_ represents the characteristic reflection of montmorillonite (JCPDS card NO. 29–1499). The peaks at 2θ = 20.9°, and 26.6° of quartz (JCPDS card NO. 46–1045) and dolomite are observed at 23.43° (JCPDS card NO. 36–0426). These findings indicate that the support was a typical bentonite [43]. Chemical composition of raw (YC) which is an important mineral resource of Moroccan was given in Table 2, indicates that the predominant oxide is silica followed by alumina Al_2_O_3_ associated with the material phases. In addition, it may be concluded referring to the highly intensive d_101_ features of the sample that Al is highly available in the octahedral centers of YC [44]. The high Mg and Ca contents of the raw Yellow clay (Table 2) illustrate the significant amount of Mg^2+^ and Ca^2+^ contribution from dolomite to the framework Mg and interlayer Ca cations [45]. The diffraction peaks of CuO were detected in Figure 1b around 35.43°, 38.66°, 48.7° and 61.58° corresponded to the d_002_, d_111_, d_-202_ and d_113_ respectively (JCPDS card NO. 01-089-2530) [29,31], and the characteristic peaks of NiO were detected in Figure 1c and d at 2θ = 37.2°, 43.3° and 62.87, which ascribed to the plane d_111_, d_200_, d_220_ of the cubic phase NiO, respectively (JCPDS card NO. 47–1049) [46] for a mixture of copper and nickel oxide are seen at 62.8° with the d_220_ to the crystalline structure of (Cu–Ni)O Figure 1c. The crystallite size of CuO and NiO calculated using the Scherrer formula [8] was 11.47 and 12.57 nm respectively.

**Table 2:** Chemical composition of raw yellow clay (YC).

Constituent	SiO_2_	Al_2_O_3_	Fe_2_O_3_	CaO	MgO	Na_2_O	K_2_O	TiO_2_	MnO	P_2_O_5_	L.O.I ∗
Constituent (%)	58.92	25.21	3.63	1.31	2.37	1.89	0.82	0.20	0.01	0.04	5.60

**L.O.I:** Loss on Ignition.

Some physicochemical properties of the raw YC and the other three catalysts are reported in Table 3. As it can be seen, the samples showed an increase in the nickel loading results and a decrease in specific area using both BET and Langmuir surface area methods, relative low surface area probably associated to the micro-pore filled intrinsic impregnation procedure used, due to the deposition of the Ni and Cu hydroxyl nitrate [47, 48, 49]. Which is in a good agreement with the experimental study given CWPO reaction, because the removal efficiency of CAF with adding H_2_O_2_ to the solutions was used by the oxidation not by adsorption. The final catalysts were also analyzed by ICP in order to determine the Cu and Ni contents in the prepared catalysts. The results show that the catalysts formed are CuNi10-YC, CuNi11-YC and CuNi01-YC with Cu–Ni 87.41–0.01; 45.05–43.4 and 0.01–85.38 mg.L^−1^ respectively. These results are in agreement with the expected stoichiometric ratio of Cu to Ni used Table 3.

**Table 3:** Langmuir constants for adsorption of methylene blue dye on catalysts and ICP.

Catalysts	Longmire Specific surface area (m^2^.g^−1^)	BET (m^2^.g^−1^)	ICP			
			Cu (mg.L^−1^)	Ni (mg.L^−1^)	Leached metal ions (mg.L^−1^)	
					Cu (mg.L^−1^)	Ni (mg.L^−1^)
Raw YC	74.39	61.02	<0.01 87.41 45.05	<0.01 <0.01 43.40	**--** 2.05 0.78	**--** **--** 8.11
CuNi10-YC	69.12	57.81				
CuNi11-YC	63.54	53.96				
CuNi01-YC	58.06	51.57	<0.01	85.38	**--**	15.43

**Figure 2:**
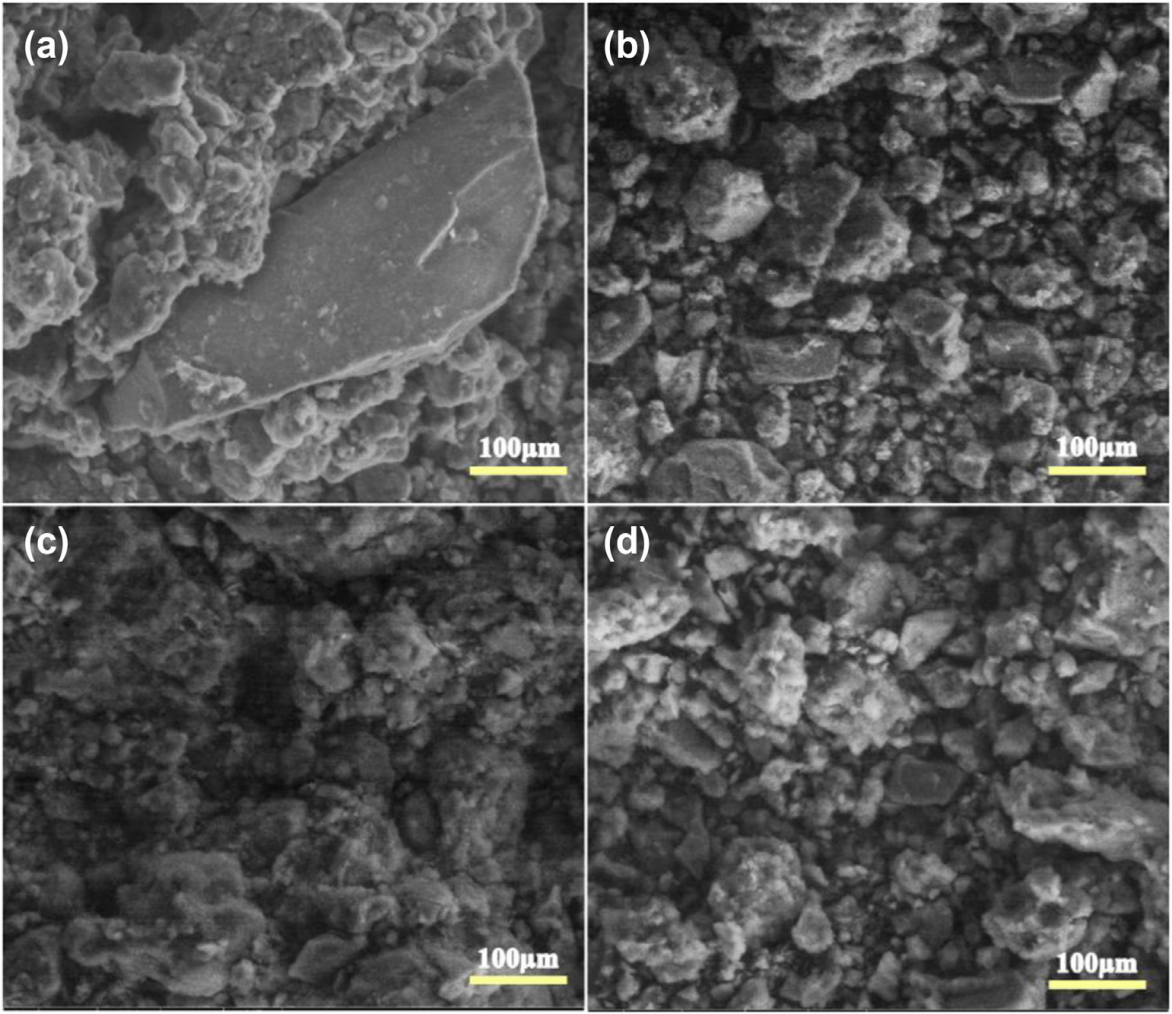
SEM micrographs of support and CuNi-YC fresh catalysts: (a) support and (b) CuNi 1:0 catalyst; (c) CuNi 1:1 catalyst; (d) CuNi 0:1 catalyst.

Figure 2 provides a SEM micrograph, which illustrates the morphology of YC, CuNi10-YC, CuNi11-YC and CuNi01-YC catalysts. Figure 2. a shows YC structure while it was being organized into aggregated patches of various agglomeration with different sizes. The morphological appearance of the following Cu:Ni weight ratios: 1:0, 1:1 and 0:1 catalysts is illustrated in Figure 2b, c and d respectively. The samples appear to have smaller agglomerates comparing to YC alone. The most probable cause of the observed changes in the morphological appearance of the catalysts may be due to the strong immobilized CuO and NiO nanoparticles on natural yellow clay support. These obtained results are already confirmed by XRD analysis and the same results was reported by Alakhras et al [50] when the Titania (TiO_2_) loaded zeolite material. However, the sizes of individual particles in the agglomerate cannot be clearly seen in these micrographs.

### 3.2 Statistical analysis

We have adopted the response surface methodology through Box–Behnken design for investigating the statistic analyze and optimizing the impact of the three factors (H_2_O_2_ dosage, impregnated copper (%) and temperature) screened concerning the degradation of CAF using the local clay as a support. The number of experiments used in this study are 17, calculated using the following formula (6), where k = 3 is the number of the studied factors and C_0_ = 5 is the number of central points (numbers 13–17), the (%) degradation of CAF in this study was observed in the range of 40–86 % (Table 4) [51]. In addition, the regression model was observed in terms of the three factors which are expressed through the following second-order polynomial Eq. (7).

(6) $$N=2\times k\times \left(k-1\right)+C_{0}$$

(7) $$\text{Y }\left(\text{\%}\right)=69.8-2.375X_{1}+16.125X_{2}+3.75X_{3}-11.025X_{1}^{2}-2.525X_{2}^{2}-1.775X_{3}^{2}-1.25X_{1}X_{2}+0.0X_{1}X_{3}+0.5X_{2}X_{3}$$

**Table 4:** Experimental design and responses results of caffeine conversion.

Experiment no	[H_2_O_2_]	Impregnated Copper	Temperature	Conversion
	(mol.L^−1^)	%	°C	%
1	0.082	0	40.00	43.00
2	0.246	0	40.00	40.00
3	0.082	10.00	40.00	75.00
4	0.246	10.00	40.00	67.00
5	0.082	5.00	20.00	54.00
6	0.246	5.00	20.00	50.00
7	0.082	5.00	60.00	64.00
8	0.246	5.00	60.00	60.00
9	0.164	0	20.00	46.00
10	0.164	10.00	20.00	80.00
11	0.164	0	60.00	50.00
12	0.164	10.00	60.00	86.00
13	0.164	5.00	40.00	70.00
14	0.164	5.00	40.00	70.00
15	0.164	5.00	40.00	70.00
16	0.164	5.00	40.00	69.00
17	0.164	5.00	40.00	70.00

The results of ANOVA used for checking the validation of this work's model are presented in Table 5. Certainly, the p-value corresponding to caffeine conversion is an extremely low probability (p-value less than 0.05) indicating that the model is highly significant, [33]. In addition, the determinant regression coefficient (R = 0.990 and the adjusted regression coefficient (adjusted R = 0.976) for both responses are closer to 1. Furthermore, the larger the ratio and the lower the p-value are, the more significant the corresponding parameter will be in the regression model. Therefore, these results show that the models fit well and the experimental data could be well modeled for both responses [33, 41].

**Table 5:** Analysis of variance for the regression models of caffeine conversion.

Source of variation	Sum of square	Degree of freedom	Mean of square	Ratio	Significance
Regression	2.82457E+0003	9	3.13841E+0002	1569.2042	<0.01 ∗∗∗
Residuals	2.95500E+0001	7	4.22143E+0000		
Validity	2.87500E+0001	3	9.58333E+0000	47.9167	0.136 ∗∗
Error	8.00000E-0001	4	2.00000E-0001		
Total	2.85412E+0003	16			

Besides, the obtained p-value implies the importance of each factor in obtaining an efficient removal of CAF. Therefore, it can be seen in Table 6 that all the model's terms such as linear (x_1_, x_2_ and x_3_) quadratic (x_1_^2^, x_2_^2^ and x_3_^2^) and interactive effects (x_1_ x_2_, x_2_ x_3_ and x_1_ x_3_) are statistically significant. Hence, the results show that the catalyst has the most significant effect on the caffeine conversion.

**Table 6:** Estimated value of regression coefficients and their significance in the quadratic model for caffeine conversion.

Coefficient	Estimate coefficient	Inflation factor	Standard deviation	t.exp.	Confidence level (%)	Signification
b0	69.800		0.2	349.00	<0.01	∗∗∗
b1	-2.375	1.00	0.15811388	-15.02	0.0114	∗∗∗
b2	16.125	1.00	0.15811388	101.98	<0.01	∗∗∗
b3	3.750	1.00	0.15811388	23.72	<0.01	∗∗∗
b1-1	-11.025	1.01	0.21794495	-50.59	<0.01	∗∗∗
b2-2	-2.525	1.01	0.21794495	-11.59	0.0317	∗∗∗
b3-3	-1.775	1.01	0.21794495	-8.14	0.124	∗∗
b1-2	-1.250	1.00	0.2236068	-5.59	0.502	∗∗
b1-3	0.000	1.00	0.2236068	0.00	100.0	
b2-3	0.500	1.00	0.2236068	2.24	8.9	

T.exp is the value of variables determined by Student's test.

∗∗∗Significant for 0.0001 < p value <0.001.

∗∗Significant for 0.001 < p value <0.01.

∗Nonsignificant for p value >0.05.

**Figure 3:**
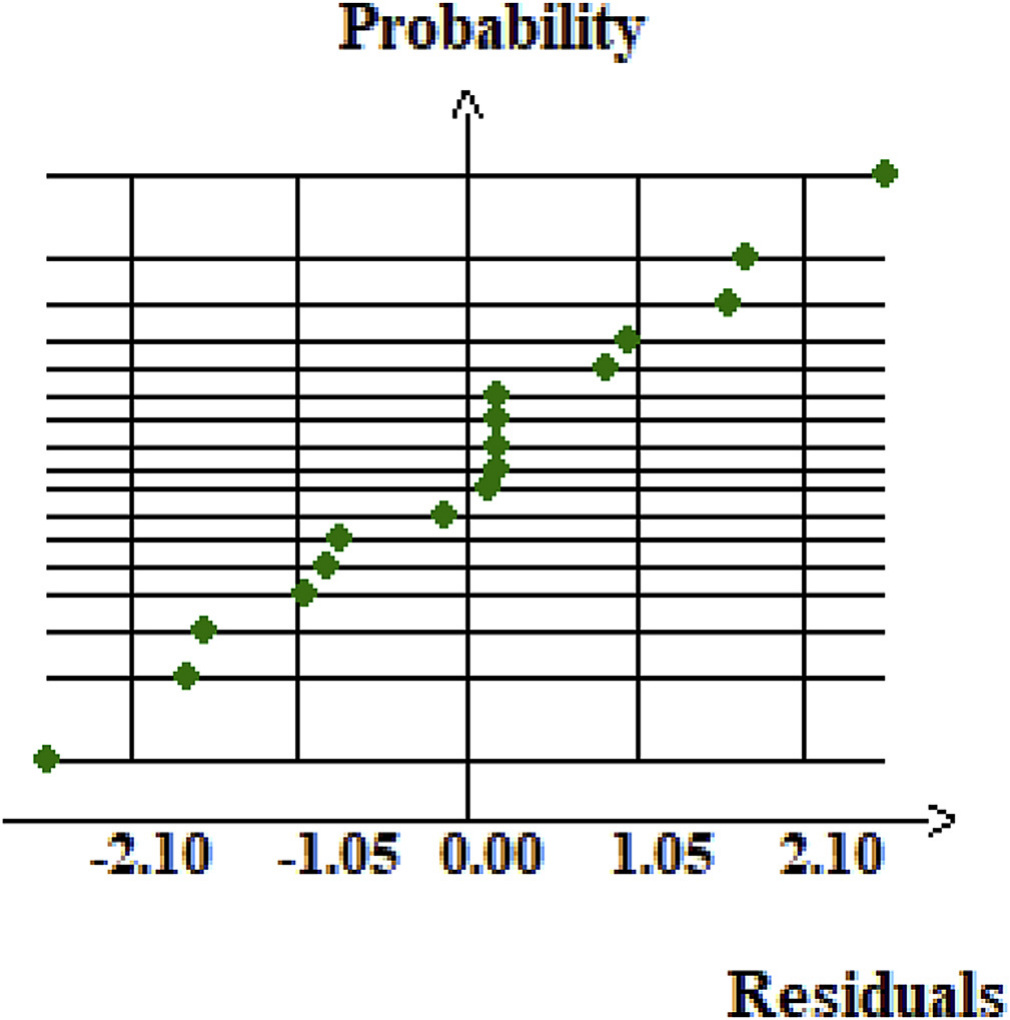
Graphic study of the residues of the response (caffeine conversion).

Figure 3 displays the residuals plots versus the normal plot probability of the responses residuals, which show a random distribution. This confirms the adequacy of the models. Most points relatively follow the straight-line x = y. This indication is in accordance with the previous results obtained in Table 5.

### 3.3 Response surface plots of effect of the three parameters studied

**Figure 4:**
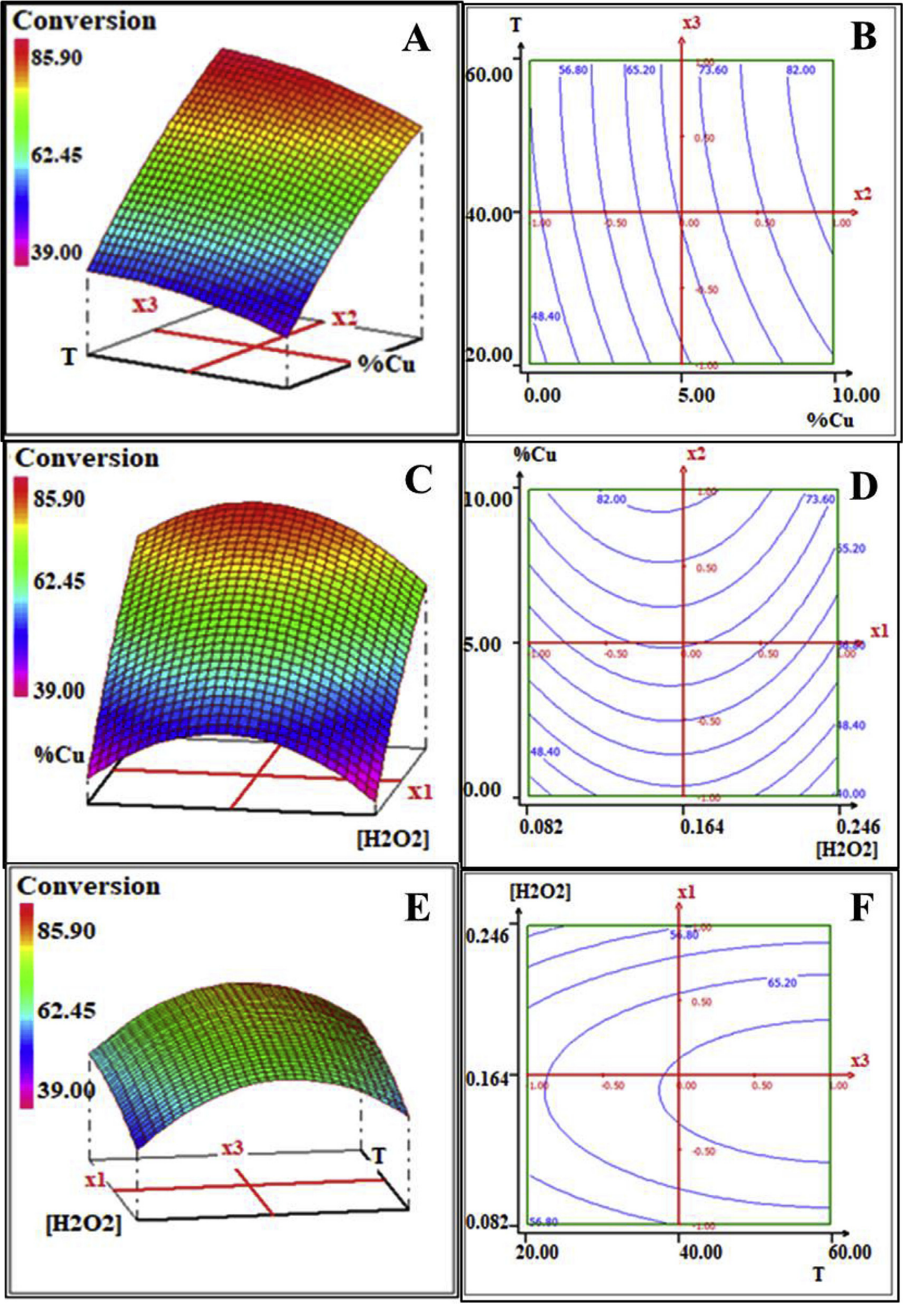
2D and 3D plots for effect of (A,B) impregnated copper and temperature (C,D) H_2_O_2_ concentration and impregnated copper (E,F) temperature and H_2_O_2_ concentration.

Figure 4 shows the 3D response surface plots and there matching 2D contour plots corresponding to the effect of the three parameters on the removal efficiency of CAF using 1 g.L^−1^ of catalyst.

The results show that the 3D and 2D plots related to impregnated copper and temperature effects, at 0.082 mol.L^−1^ of H_2_O_2_. It is shown in Figure 4A and B that the removal efficiency of CAF increased along with the increase of reaction temperature at higher amount of impregnated copper. When the temperature's reaction reached 60 °C, the conversion of CAF efficiency was the highest (>80%), which is in a good correlation with the Arrhenius theory of the temperature's positive influence on the rate constant, by enhancing the feasibility of the degradation process. However, the removal efficiency decreases if the temperature continued to rise, because the temperature was too high making the faster decomposition of H_2_O_2_, and the utilization rate of H_2_O_2_ was greatly reduced according to Eq. (8) [51, 52].

(8) 2H_2_O_2_ → 2H_2_O + O_2_

The 3D and 2D plots related to H_2_O_2_ and catalyst copper content, at 20 °C temperature. It is shown in Figure 4C and D that the maximal conversion of CAF (>80%) was achieved at higher amount of impregnated copper and medium volume of H_2_O_2_. However, at the lowest impregnated copper and an excess of H_2_O_2,_ the CAF conversion reached its minimum. In addition, it was also obvious that CuNi10-YC was the most efficient catalyst and has the active phase for hydrogen peroxide as well as CAF molecules: the catalyst has the activation sites for H_2_O_2_ and caffeine. However, when nickel was incorporated in clay matrix, it led to a decrease in the CAF conversion [29]. The results could explain that the presence of nickel reduces the Langmuir surface and leads to a distortion of the clay matrix. It was more important that although the TOC value decreased until 27% in the presence of nickel.

CuNi01-YC catalyst was used in the following experiments. The effect of hydrogen peroxide's reaction and temperature treatment performance of CAF wastewater were investigated. The removal efficiency of CAF with different amount of peroxide dosage was illustrated in Figure 4E and F. Hydrogen peroxide has the same behaviour as temperature on the removal efficiency of CAF. In fact, the degradation rate reached the maximum value, when peroxide dosage was ≤0.164 mol.L^−1^. However, once the initial concentration of H_2_O_2_ is higher than 0.164 mol.L^−1^, the removal efficiency drops down. This might be ascribed to the scavenging effect of excess H_2_O_2_ on the active hydroxyl radical HO^•^ to produce HO_2_^•^ less active as illustrated by (equation: 9, 10, 11) [51,52].

(9) H_2_O_2_**→** 2HO^•^

(10) HO^•^ + H_2_O_2_**→** HO_2_^•^ + H_2_O

(11) HO_2_^•^ + HO^•^ → O_2_ + H_2_O

### 3.4 Optimization of factors for caffeine conversion

The CAF conversion reached its maximal 86.16 %, when H_2_O_2_ dosage was equal to 0.15 mol.L^−1^, copper impregnated (10 %) and temperature value attained 60 °C Table 7.

**Table 7:** Predicted and experimental values of caffeine conversion under optimal conditions.

Variable	Value	Factor	Value	Caffeine removal (%)	
X1	-0.168094	[H_2_O_2_]	0.150	optimum combination of the model 86.16	Experimental validation of the model 87.03
X2	0.999844	%Cu	10.00		
X3	0.988281	T°	59.77		

**Figure 5:**
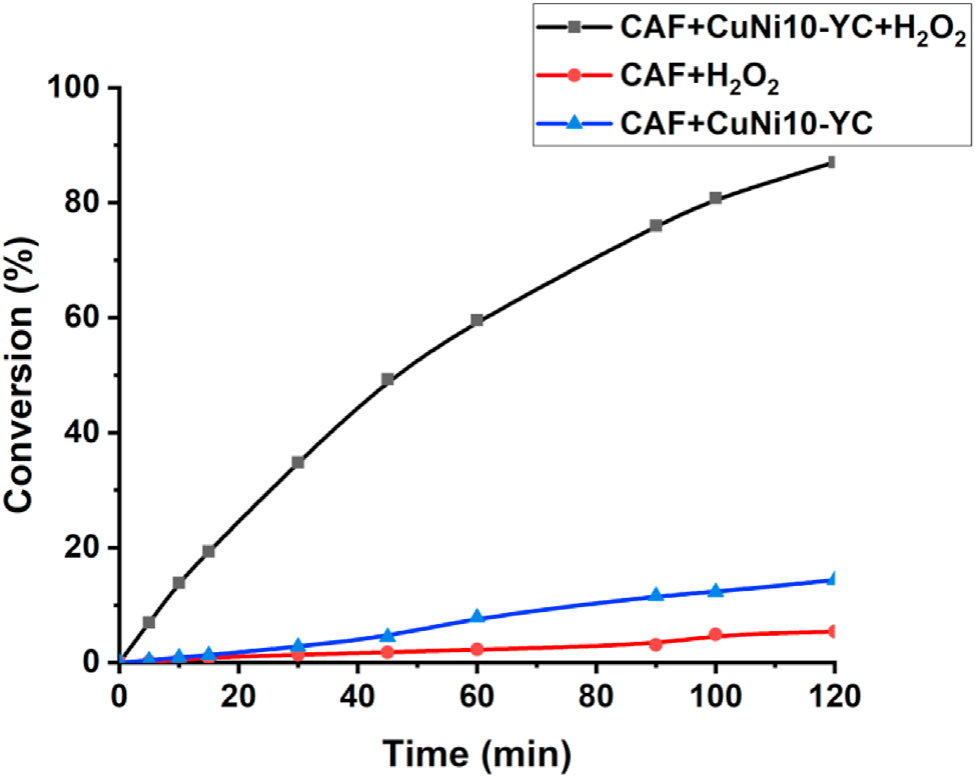
Catalytic activity of CuNi10-YC in the CAF oxidation under optimumconditions, [H _2_O_2_] = 0.15 mol.L^−1^, m_cat_ = 1 g.L^−1^, T = 60 °C and [CAF] = 40 mg.L^−1^.

Figure 5 shows the CAF conversion under optimum conditions after using Box-Behnken design. It was seen that CAF could be mostly (87.03%) degraded with 68.85 % as the final value of TOC mineralization after 120 min in the coexisting system of CuNi10-YC and H_2_O_2_, which is in a good agreement with the predicted value given by the Box-Behnken model. On the contrary, in the presence of CuNi10-YC or H_2_O_2_ alone, the CAF conversion after 120 min was only 14.39 % and 5.4 %, respectively. The low percentage of caffeine decomposition in the presence of the peroxide alone, can be attributed to the deficient homogenous decomposition H_2_O_2_ in hydroxyl radical [53]. In addition, the effect of both metals copper and nickel impregnated on yellow bentonite clay ameliorate the oxidation efficiency of the raw clay and as we can see in Table 3 the amount of copper (CuNi10YC) leached after 120 min of reaction, was lower than nickel (CuNi01YC). Accordingly, CuNi10YC catalyst is expected to exhibit high CAF oxidation and stability and not losing the Cu metal by leaching in the medium. When both metals (CuNi11YC) were impregnated, the CAF conversion decreases which can be explained by the decrease in the catalyst's surface area (CuNi11YC), the high amount of leached nickel leads to a distortion of the clay matrix.

### 3.5 Consecutive cycles of photocatalytic runs

**Figure 6:**
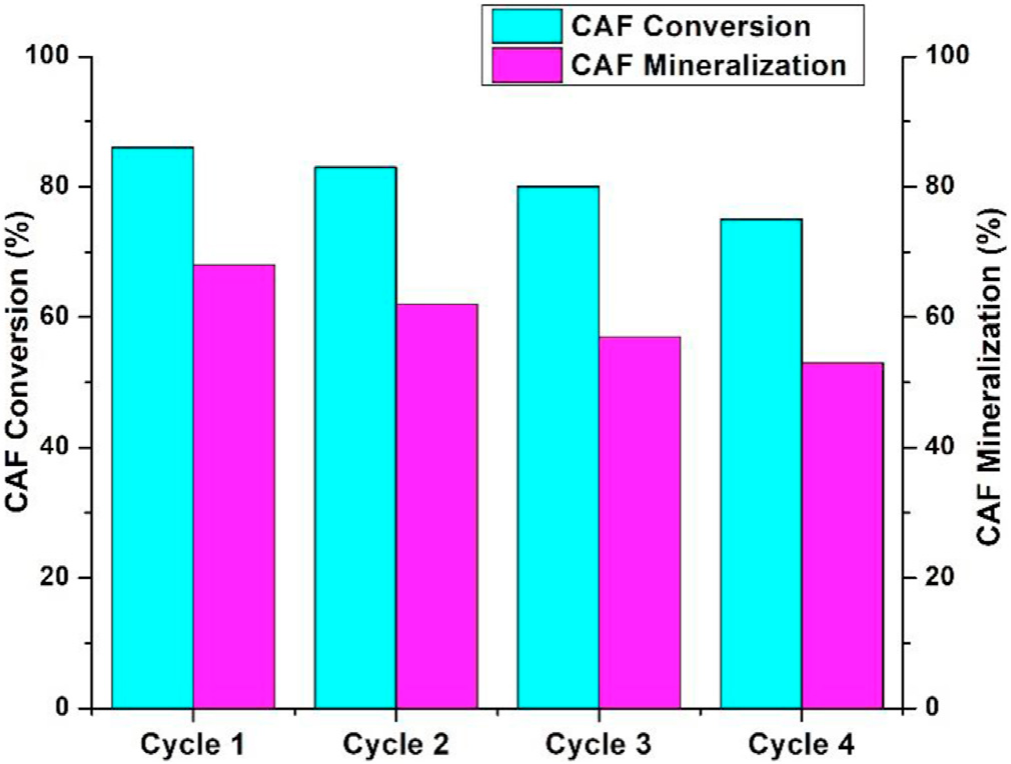
Conversion and mineralization percentages during the consecutive's cycles for the CAF conversion using CuNi10-YC catalyst.

Additionally, to the high efficiency of the eco-friendly synthesized catalyst CuNi10-YC in degrading CAF molecule from wastewater. The stability plays an important role in checking out the best photocatalyst from others. Figure 6 shows the degradation percentages of the CAF solution (40 ppm) after 120 min of oxidation, it also shows that an important conversion is obtained (~87%) in each reuse. A slight decrease in the mineralization percentages was obtained from the TOC values during the four recycling tests. This indicates that the catalyst remains stable in successive reuses. The insignificant reduction in oxidation performance observed could be caused by the inevitable loss of the catalyst mass during washing and centrifugation processes.

### 3.6 Toxicity evaluation of CAF degradation

**Figure 7:**
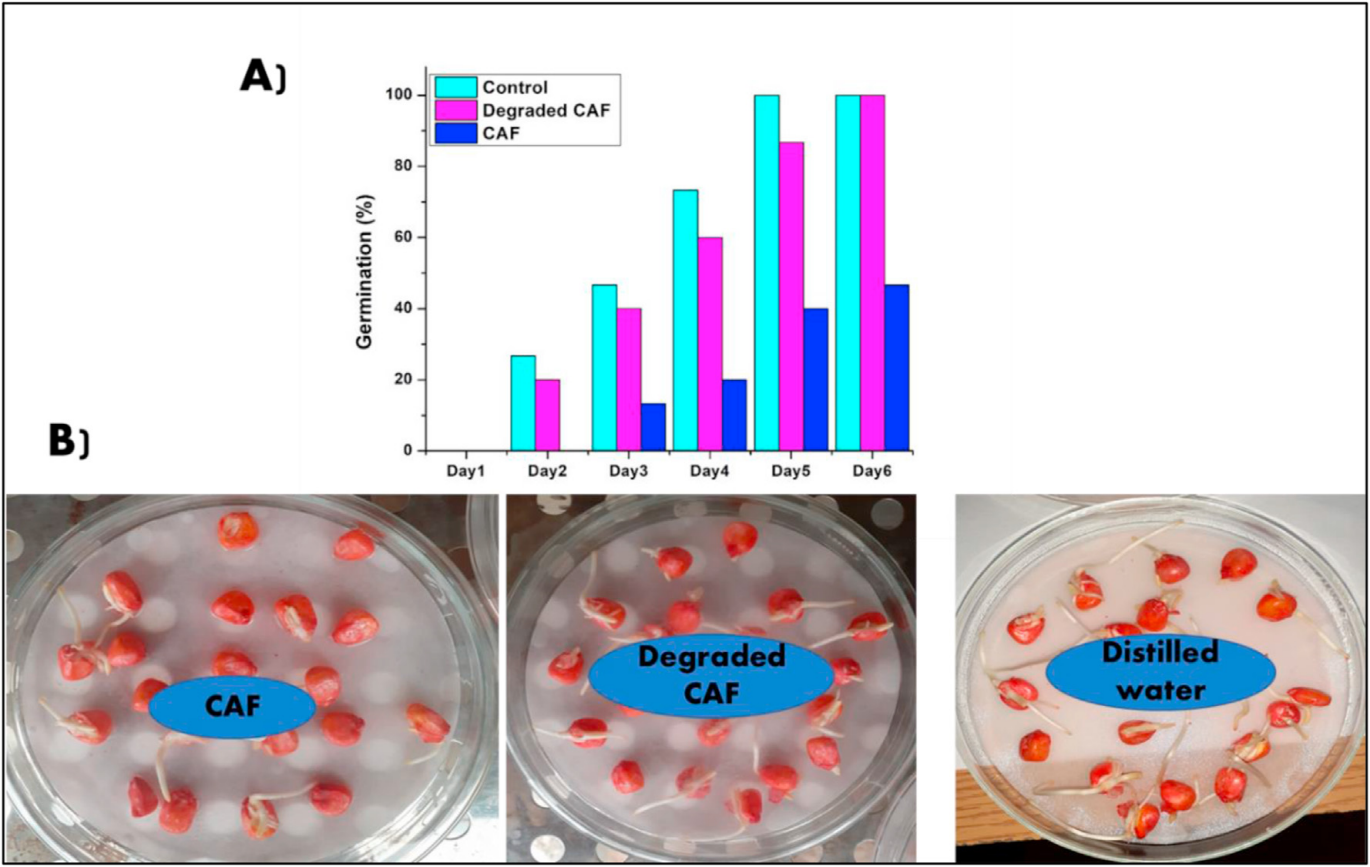
Germination of corn kernels in CAF and degraded CAF.

For germination test, populations of corn kernels were exposed to a pollutant (CAF molecule) dissolved in distilled water, its toxicity was estimated to evaluate the toxic effects, such as an inhibition of the germination rate (Figure 7). It was observed that in only 6 days the germination of corn kernels in distilled water (control or Blank) was totally (100 %). On the other hand, the germination rate was lower for untreated aqueous solutions containing CAF, which did not exceed 50 % (Figure 7A). However, the germination rate in the presence of catalyst (CuNi10-YC) CAF aqueous solutions reached 100 % in 6 days (Figure 7A and B). According to the caffeine conversion and TOC results, the CuNi10-YC catalyst has a significant ability to degrade CAF molecules from wastewater.

## 4 Conclusion

The objectives of this work were successfully achieved, through incorporating copper/nickel into yellow clay prepared using wet impregnation method, and characterizing it by XRD, XRF, ICP, SEM, BET and Langmuir surface area analysis. The CWPO test for degradation of caffeine has successfully confirmed the usefulness of the modified clay as a catalyst. Thanks to Box-Behnken's design, the optimal conditions for degradation were selected. It has revealed that the highest CAF conversion was achieved was 86.16 % using CuNi10-YC catalyst. A feasible CAF conversion reaction system could be applicated as follows: 1 g.L^−1^ of catalyst, temperature of 60 °C, 40 mg.L^−1^ of CAF and time of 120 min, where CAF could be completely oxidized, and the final value of TOC mineralization more than 68.85%. This study has reported relevant and original results demonstrating that CWPO using CuNi-YC processes could be a cost-effective, stable and efficient alternative treatment for the removal of caffeine, since the germination test has given a positive index of good degradation. Another advantage of using this modified clay is its possibility of removing different type of organic compounds. Therefore, it would be interesting to continue testing on other persistent organic pollutants, and why not testing a real wastewater not only batch processes, but also column processes in a pilot scale.

## Declarations

### Author contribution statement

Ouissal Assila: Conceived and designed the experiments; Performed the experiments; Wrote the paper.

Morad Zouheir, Karim Tanji: Conceived and designed the experiments; Analyzed and interpreted the data; Wrote the paper.

Redouane Haounati: Contributed reagents, materials, analysis tools or data; Wrote the paper.

Farid Zerrouq: Analyzed and interpreted the data; Wrote the paper.

Abdelhak Kherbeche: Analyzed and interpreted the data; Contributed reagents, materials, analysis tools or data; Wrote the paper.

### Funding statement

This research did not receive any specific grant from funding agencies in the public, commercial, or not-for-profit sectors.

### Data availability statement

No data was used for the research described in the article.

### Declaration of interests statement

The authors declare no conflict of interest.

### Additional information

No additional information is available for this paper.
